# Effects of Massage Therapy in Breast Cancer Survivors with Mastectomy: Systematic Review

**DOI:** 10.3390/cancers17122023

**Published:** 2025-06-17

**Authors:** Juan Rodríguez Mansilla, Ana Sánchez Díaz, Blanca González Sánchez, María del Valle Ramírez-Durán, Elisa María Garrido Ardila, María del Carmen Cilleros Sánchez, María Jiménez Palomares

**Affiliations:** 1 ADOLOR Research Group, Department of Medical-Surgical Therapy, Medicine Faculty, Extremadura University, 06006 Badajoz, Spain; jrodman@unex.es (J.R.M.); egarridoa@unex.es (E.M.G.A.); mariajp@unex.es (M.J.P.); 2 Clínica Fisioterapia Belén Escudero, Campanario, 06460 Badajoz, Spain; anaisabelsd11@gmail.com; 3 Department of Nursing, University Center of Plasencia, University of Extremadura, 10600 Plasencia, Spain; valleramirez@unex.es; 4 Department of Medical-Surgical Therapy, Nursing and Occupational Therapy Faculty, Extremadura University, 10003 Cáceres, Spain; ccilleros@unex.es

**Keywords:** mastectomy, breast neoplasms, massage, manual lymphatic drainage, myofascial release

## Abstract

Background: Mastectomy, a common treatment for breast cancer, often leads to complications such as pain, fibrosis, restricted mobility, lymphedema, reduced strength in the affected arm, and emotional distress. Non-pharmacological therapies, including massage therapy, offer a holistic approach to managing these symptoms. The aim of this study was to analyze the effects of massage therapy on the symptomatology in women post-mastectomy for breast cancer. Methods: A systematic review was conducted following PRISMA guidelines. Databases including PubMed, Cochrane, PEDro, Dialnet, Science Direct, and Scopus were searched for relevant studies published in English or Spanish over the past 16 years. The search was conducted in March 2025. Inclusion criteria encompassed controlled and uncontrolled clinical trials, quasi-experimental studies, retrospective analyses, and secondary trial analyses involving women aged 45–64 who received massage therapy as a complementary treatment. Results: Twenty-six studies involving 1522 participants were included. Interventions assessed were manual lymphatic drainage, myofascial release, foot massage, classical massage, and the Cyriax technique. The key findings demonstrated significant benefits, including improved range of motion, reduced arm circumference and lymphedema volume, enhanced quality of life, and increased relaxation. Conclusions: According to the results of this systematic review, massage therapy interventions can have a positive impact on the symptomatology in women post-mastectomy for breast cancer and may represent a suitable complementary approach to post-mastectomy breast cancer treatment.

## Simple Summary

After breast cancer surgery (mastectomy), many women experience pain, swelling, stiffness, and emotional distress, which can make recovery difficult. Massage therapy—such as lymphatic drainage or gentle muscle techniques—has been suggested as a natural way to help reduce these problems. This study reviewed existing research to see how well massage therapy works for improving movement, decreasing arm swelling, and enhancing well-being in breast cancer survivors. By analyzing 26 studies involving over 1500 women, the researchers found that massage can help increase flexibility, reduce swelling, and improve quality of life without harmful side effects. These findings are important because they support massage as a safe and helpful addition to standard cancer recovery care. This research could encourage doctors and therapists to include massage therapy in rehabilitation programs, offering women a gentle yet effective way to heal after surgery. Future studies could help determine the best massage techniques for long-term recovery.

## 1. Introduction

Breast cancer remains one of the most prevalent types of cancer globally, currently representing the second leading cause of death among women and the most frequently diagnosed type of cancer in Spain [1,2,3]. Although advancements in early detection and treatment have significantly improved survival rates, recurrence and mortality persist as critical challenges [2].

The clinical presentation of breast cancer is often insidious, with many patients remaining asymptomatic during the initial stages of the disease; once symptoms emerge, the most common include a palpable breast mass, persistent local skin changes such as redness, skin retraction, or an orange-peel appearance, nipple eczema, axillary lymphadenopathy, unilateral arm edema, nipple retraction, and abnormal nipple discharge [2,4,5]. Beyond physical symptoms, breast cancer patients frequently experience psychological distress, including mood disorders, lifestyle disruptions, and heightened fear related to diagnosis and prognosis [6].

The primary therapeutic interventions for breast cancer include breast-conserving surgery and mastectomy, frequently combined with chemotherapy, radiotherapy, and hormonal therapy [2,3,5,7,8,9]. While surgical treatments have significantly improved survival outcomes, studies show that they might trigger some adverse effects, including chronic pain, arm edema, fibrosis, skin retraction, kinesiophobia (fear of movement), and notably lymphedema, which exhibits a higher incidence compared to other sequelae, and without early intervention can lead to functional impairment, including reduced mobility and diminished strength in the affected upper limb. Upon progression to a chronic condition, this severely impacts patients’ quality of life [3,7,8,9,10,11,12,13]. In addition to these effects, other complications may arise that affect the vascular and lymphatic systems, joints, pain levels, and neurological function [14,15]. These issues highlight the need for a comprehensive postoperative rehabilitation process that addresses this complex condition holistically, integrating not only physical concerns, but also the psychological dimension and the challenge of social reintegration [16]. The consequences of breast cancer often represent a significant life change for those affected, due to the impact on their daily activities. However, these effects can be managed and reduced through comprehensive rehabilitation programs tailored to each individual case [17].

Consequently, early postoperative rehabilitation is critical to mitigate these effects, encompassing educational strategies, stretching exercises, progressive physical activity, manual lymphatic drainage (MLD), and scar massage. In addition, massage therapy is widely regarded as a highly effective intervention for alleviating symptoms and improving functional outcomes in the affected upper limb following breast cancer surgery [5,18]. This modality involves the systematic manual manipulation of soft tissues, with demonstrated efficacy in reducing pain, inflammation, nausea, fatigue, and psychological distress, including symptoms of depression and anxiety [4,10].

Among the diverse techniques employed, postoperative MLD contributes to reducing lymphatic fluid accumulation, decreasing limb volume, improving range of motion, and enhancing quality of life [11,13,19,20]. Similarly, myofascial release (MFR) therapy has been shown to restructure collagen fiber alignment, thereby optimizing tissue mobility, promoting fluid dynamics, increasing joint flexibility, and mitigating pain perception [7,8,9,12,18]. Both techniques are considered safe in the context of potential residual tumor cells when administered by trained physiotherapists with expertise in oncology rehabilitation.

MFR is a manual therapy treatment that involves the application of low-load, long-duration mechanical forces specifically directed to manipulate the myofascial complex. It consists of applying slow, sustained pressure (120–300 s) to restricted fascial layers, either through direct (direct MFR technique) or indirect (indirect MFR technique) approaches [21].

Accordingly, the objective of this systematic review was to analyze the effects of massage therapy on the symptomatology of women with breast cancer who have undergone mastectomy.

## 2. Materials and Methods

### 2.1. Study Design

This systematic review was conducted in accordance with the PRISMA recommendations [22]. The review protocol is available on PROSPERO (registration number: CRD42024547433).

In order to identify relevant studies, a comprehensive search was carried out in the following databases: PubMed, Cochrane, PEDro, Dialnet, ScienceDirect, and Scopus.

### 2.2. Search Strategy

The literature search was conducted using the following key terms: mastectomy, breast neoplasms, massage, manual lymphatic drainage, and myofascial release. To ensure a comprehensive retrieval of relevant studies, equivalent Spanish terms—mastectomía, cáncer de mama, masaje, masoterapia, drenaje linfático manual, and liberación miofascial—were also employed in databases requiring Spanish-language queries. These keywords were strategically combined using the Boolean operator “AND” to refine search results and enhance precision. The specific search strategies applied in each scientific database are detailed in Table 1.

### 2.3. Eligibility Criteria

Eligibility criteria were defined following the PICOS framework (Population, Intervention, Comparison, Outcome, Study design), and were related to the effects of massage therapy in women who had undergone mastectomy.

Inclusion criteria:

1. Type of participant: Female participants aged 45–64 years who had undergone mastectomy.
2. Type of intervention: Interventions involving massage therapy as an adjuvant treatment.
3. Type of study: Randomized controlled trials (RCTs), uncontrolled trials, secondary analyses of clinical trials, quasi-experimental studies, and retrospective analyses.
  - Studies published within the last 16 years.
  - Publications in English or Spanish.
  - Research published in the last 16 years to assess the most recent advancements in massage therapy as an adjuvant treatment and to update the available scientific evidence on this topic.
4. Outcome measures: Any outcome measure assessed with a standardized or validated assessment tool.
  Exclusion criteria:
  - Study protocols, systematic reviews, and meta-analyses.
  - Studies with fewer than 4 participants.
  - Interventions not classified as massage therapy techniques.

### 2.4. Study Selection

An initial screening of identified studies was performed to evaluate their relevance to the research objectives. This preliminary screening was conducted through abstract review, excluding those that did not meet the established criteria.

**Figure 1:**
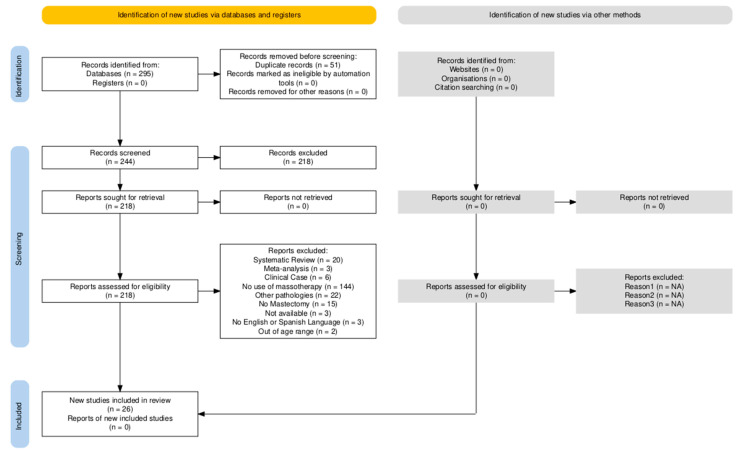
PRISMA diagram.

Full-text articles that met the selection criteria were subjected to comprehensive evaluation. Two independent reviewers examined all potentially eligible publications. While inter-rater reliability was not quantitatively assessed, any discrepancies in study selection were resolved through consensus-based discussion (see Figure 1 for PRISMA flow diagram).

From the selected studies, we systematically extracted the following information: sample characteristics, study design, description of the intervention and control/experimental groups, outcome measures, and study results.

These data were organized into a standardized evidence table. The same dual-reviewer system was implemented for both data extraction and methodological quality assessment. Independent evaluation was maintained, with disagreements resolved by consensus.

### 2.5. Methodological Quality Analysis

Methodological quality was assessed using the PEDro scale [23], an 11-item tool (scored yes/no) yielding a 0–10 score (Item 1 is not scored as it assesses external validity); higher scores indicate better quality, categorized as excellent (9–10), good (6–8), fair (4–5), or poor (≤3). Items 2–9 evaluate internal validity (randomization, blinding, attrition), while Items 10–11 assess statistical reporting adequacy. A trained reviewer conducted all quality assessments to ensure consistency in the application of the scoring criteria.

### 2.6. Risk of Bias Analysis

Risk of bias was assessed for each included study using seven bias domains: (1) random sequence generation (selection bias), (2) allocation concealment (selection bias); (3) participant/personnel blinding (performance bias); (4) outcome assessor blinding (detection bias); (5) incomplete outcome data (attrition bias); (6) selective reporting (reporting bias); and (7) other potential biases. Assessments were conducted by a single reviewer.

## 3. Results

The literature search was conducted in March 2025. A total of 295 studies were retrieved across all databases. The PRISMA flow diagram (Figure 1) illustrates the study selection process. After removing duplicate records, 245 records were screened. Ultimately, 26 studies were included in this review.

The key characteristics of the included studies are summarized in Table 2. The total sample size across all studies comprised 1522 participants, with notable variability in individual study sample sizes. Six studies [4,11,24,25,26,27] enrolled more than 100 participants each, whereas eleven studies [3,7,8,10,12,19,28,29,30,31,32] had sample sizes of 40 or fewer.

The review encompassed 23 clinical trials [3,4,7,10,11,12,13,19,20,24,25,26,27,28,29,30,31,32,33,34,35,36,37,38], 4 of which were pilot studies [10,31,33,37]. Additionally, one secondary analysis of a clinical trial [9], one pre–post experimental study [8], and one retrospective analysis [39] were included. Due to heterogeneity in the study designs, not all investigations incorporated a control group, restricting direct comparisons between interventions, alternative techniques, or placebo conditions.

Among the studies with control groups, comparison interventions varied. These included compression therapy [25] and postural guidance [35], though the most frequently used comparators were standard/routine healthcare [3,4,13,31,33] and physical exercise [11,20,24,26,30,32,36]. Particularly, Kim et al. [12] employed a distinct methodology: both the control and experimental groups received identical treatments—30 min of myofascial release (MFR) therapy followed by 30 min of complex decongestive therapy (CDT)—but in reverse sequences (Group A: MFR → CDT; Group B: CDT → MFR).

**Table 2:** Characteristics of the studies included in this review.

Authors	Sample, Mean Age	Study Design and Intervention	Treatment and Follow-up Period	Type of Massage Therapy	Assessment Tools	Main Findings
Arinaga Y, Piller N, Sato F et al. [33]	*N* = 43 (CG: 21; EG: 22), Mean age ≥ 20	Randomized controlled pilot study. CG received standard hospital care; EG participated in a self-care program for breast cancer-related lymphedema (BCRL).	EG performed a 10-min daily holistic self-care routine (radio Taiso: 3 min; arm exercises: 1 min; central lymphatic drainage: 1 min; hydration: 3 min). CG received standard care. Follow-up at baseline, week 1, and months 1, 3, and 6.	Central MLD at subclavian drainage points during bathing; skin care with milk lotion using MLD techniques toward thoracic duct.	L-Dex, Relative Edema Volume (REV), Relative Volume Change (RVC), Transepidermal Water Loss (TEWL), skin induration, BCRL symptoms, SF-8, QOL.	No significant differences in extracellular fluid or skin induration. Significant differences observed in hand measurements (REV and RVC), but not in the arm. TEWL differed significantly in breast (EG: *p* = 0.043), hand, and arm. EG showed greater improvement in BCRL symptoms and quality of life.
Baklaci M, Eyigör S, Tanlgör G et al. [34]	*N* = 74 Mean age: 56 ± 10.74	Descriptive observational clinical trial. All participants enrolled in complex decongestive therapy (CDT).	Program included patient education, skin care, 20 min exercises, 30 min walking, 45 min MLD, and compression bandaging, at least 5 days/week in two phases. Weekly evaluations.	Patients were taught to perform slow, rhythmic MLD on the affected limb.	Handgrip dynamometry (JAMAR), volumetric measurements.	Volume decreased by 46%, resulting in significant inter-arm difference (*p* < 0.01), which persisted. No significant improvement in handgrip strength.
Xiong Q, Luo F, Zhan J et al. [11]	*N* = 104 (EG1: 52, EG2: 52), Mean age: 51.9 ± 8.0	Randomized controlled trial (RCT). EG1: regular functional exercise; EG2: MLD with rehabilitation and functional exercise.	EG1 began active exercise day 1 post-op, 4×/day, 20 min each. EG2 received 1–2 daily MLD sessions (20–25 min), acupressure, and compression bandaging. Evaluations at pre-op, day 5, months 1–3.	MLD applied from central to adjacent drainage, anastomotic, and edematous zones.	Goniometry for shoulder ROM; arm circumference measured via tape.	EG2 showed significant ROM improvement over EG1 (*p* < 0.05); no significant differences in arm circumference. BCRL risk increased over time.
Uzkeser H, Karatay S, Erdemce B et al. [19]	*N* = 31 (G1: 15; G2: 16), Mean age: 37–75	RCT. G1: CDT (MLD, compression bandages and garments, exercises). G2: CDT with intermittent pneumatic compression pump.	All groups treated 5 days/week for 3 weeks. Evaluations: baseline, end of treatment (week 3), and 1 month post-treatment.	MLD in G1 only.	Measuring tape, water immersion method, ultrasonography for dermis thickness, VAS scale.	Significant volume reduction in both groups during treatment. At 7 months, only G2 maintained improvements. Circumference and dermal thickness improved only in G1. No significant intergroup difference in VAS, though pre–post improvements were seen.
Guerrero R, das Neves L, Guirro R et al. [28]	*N* = 16 (G1: 8; G2: 8), Mean age: 64 ± 11.44	RCT. G1: MLD. G2: MLD with 30° upper limb elevation.	G2 performed therapy with arm elevated to 30°. Evaluations: before, immediately after, and 30 min post-treatment.	MLD using Leduc method in supine position with or without arm elevation.	Portable continuous-wave Doppler ultrasound, measuring tape, truncated cone volume estimation method.	Significant increase in blood flow velocity with elevation. Values returned to baseline after 30 min.
Oliveira M, Gurgel M, Amorim B et al. [24]	*N* = 106 (G1: 52; G2: 53), Mean age: 55	Non-randomized controlled clinical trial. G1: MLD. G2: active exercise.	Postop education on day 1. Starting 48 h after surgery: 40 min MLD or active exercise sessions, 2/week for 30 days. Evaluations: pre-op, and 2 and 3 months post-op.	MLD techniques applied.	Goniometer, measuring tape, lymphoscintigraphy (node uptake and visualization speed).	ROM recovery was partial and similar in both groups. Significant correlation (*p* = 0.003) between initial lymphoscintigraphy scores and lymphedema development.
Martín M, Hernández M, Avendaño C et al. [13]	*N* = 58 (CG: 29; EG: 29)	RCT. CG: standard treatment (skin care, exercises, compression garments/bandaging). EG: standard care plus MLD.	Outpatient treatment for 1 month. CG: 4 weeks of multilayer bandaging, then specific exercises. EG: 4 weeks MLD + bandaging, + specific exercises. Follow-up T0, 1, 3, 6 months.	MLD starting at neck and trunk, 45–60 min/day for 2–4 weeks.	Circumference and volume via truncated cone formula, QoL questionnaires: EORTC QLQ-C30 and QLQ-BR23.	No results reported.
Dayes I, Whelan T, Julian J et al. [25]	*N* = 103 (CG: 46, Mean age: 59; EG: 57, Mean age: 61)	RCT. CG: compression garments. EG: MLD, compression bandaging, and garments.	CG wore elastic sleeves/gloves for 12 h/day. EG received 1 h MLD (5 days/week, 4 weeks), compression bandaging (23 h/day), and lifestyle counseling. Evaluations: baseline, weeks 3, 6, 12, 24, and 52.	MLD performed by Vodder/Földi-certified therapists.	SF-36, DASH, National Cancer Institute Common Toxicity Criteria.	EG showed greater mean and absolute lymphedema volume reduction, especially in long-standing cases. No DASH score differences between groups.
Cho Y, Do J, Jung S et al. [20]	*N* = 48 (G1: 24, Mean age: 50.7 ± 9.6; G2: 24, Mean age: 46.6 ± 6.8)	RCT. G1: physical therapy. G2: physical therapy + MLD.	Both groups received physical therapy 3×/week for 4 weeks: warm-up, stretching, strengthening, tissue mobilization, and MLD (30 min). Evaluated at baseline and after 4 weeks.	MLD using stationary circles, pumping, and rotary techniques.	Measuring tape, Power Track II dynamometer, digital inclinometer, EORTC QLQ-C30, QLQ-BR23, DASH, NRS.	Significant improvement in physical, emotional, and social function, fatigue, pain, arm/breast symptoms, strength, and DASH scores (*p* < 0.05). NRS and arm volume lower in G2. Lymphedema occurred only in G1.
Serra-Añó, Inglés M, Bou-Catalá et al. [7]	*N* = 24 (CG: 11; EG: 13), Mean age: 30–60	RCT. CG: MLD. EG: MFR.	Patients treated supine, arms extended, affected limb elevated 30°. Evaluations: pre-treatment, post-treatment, and 1-month follow-up.	Pilat’s MFR technique (10 min per technique, no cream).	VAS, manual goniometer, DASH, PHQ-9, FACT-B+4.	Pain improved significantly in EG (*p* < 0.05), but not in CG. ROM and function improved in both, sustained only in EG. Depression decreased only in CG. Physical well-being and FACT improved in both.
Dion L, Engen D, Lemaine V et al. [10]	*N* = 40 (G1: 20; G2: 20), Mean age: 47.7 ± 8.4	Randomized pilot study. G1: massage. G2: massage + guided meditation.	Therapy in private hospital room; 20 min massage for 3 days; G2 began with 15 min meditation. Evaluated pre-op, days 1–3, and week 3.	Swedish massage, acupressure, foot reflexology using oils.	VAS, EVA, PSS-14.	Significant improvement in VAS in both groups. G1 improved PSS-14 at week 3. No major differences between groups.
Kim Y, Park E, Lee H [12]	*N* = 30 (GA: 15, Mean age: 48 ± 8.3; GB: 15, Mean age: 47.8 ± 5.2)	Crossover RCT. GA: MFR + CDT, then placebo MFR + CDT; GB: reverse sequence.	Each group received 30 min MFR + 30 min CDT (MLD, compression, education, stretching). Evaluations: weeks 0, 4, 8 (washout), 12.	MFR applied using hand/finger to extend fascia opposite muscle direction. MLD also applied.	Measuring tape, NRS, goniometer, chest circumference, DASH, FACT-B.	Significant differences in limb volume, pain, ROM, and DASH scores (*p* < 0.05) post-intervention. Significant chest circumference and FACT-B improvement after MFR only.
Cruz-Ramos J, Cedeño-Meza A, Bernal-Gallardo J et al. [29]	*N* = 32, Mean age: 57.16 ± 11.98	Descriptive observational clinical trial. CDT with MLD.	Daily MLD sessions (40–60 min) for 5 days, followed by 24 h multilayer bandaging. Evaluations: baseline, each session, and after 5 sessions.	MLD using Vodder-Földi technique, distal to proximal.	Measuring tape, Kuhnke method, EORTC QLQ-C30.	Significant volume reduction (*p* < 0.0005), improved quality of life. Reduced fatigue, pain, dyspnea, and diarrhea. Circumference decreased only 4%.
Demirci P, Tasci S, Öztunç G [3]	*N* = 30 (CG: 15; EG: 15), Age ≥ 18	Mixed-method RCT. CG: standard care. EG: foot massage.	EG received 30 min foot massages twice weekly for 3 weeks. Evaluations: pre–post massage and weekly for 7 wks.	Foot massage using effleurage, petrissage, superficial friction.	VAS, EVA, Personal Information Form, EORTC QLQ-C30.	VAS showed significant pre–post improvement. EG had significant time-based EVA changes; CG did not. Health status improved in both, but more in EG (*p* < 0.05).
Rao M, Pattanshetty R [8]	*N* = 22, Age: 18–70	Pre–post study. MFR, stretching, and strengthening.	Four sessions/week for 3 weeks (12 total). Each included 15 min MFR + stretching (45 s) + strengthening (2 × 10 reps). Evaluations: baseline and after 12 sessions.	MFR applied to pectoralis major/minor, SCM, scalenes.	MB-Ruler v5.1 (photogrammetry), digital inclinometer, MMT, hand dynamometer, SPADI, FACT-B.	Significant improvements in posture (*p* = 0.001), spinal curvature, ROM, strength, SPADI, and FACT-B scores (*p* = 0.001).
Kasseroller R, Brenner E [39]	*N* = 62 (GA: 31; GB: 30), Mean age: 57.4 ± 8.926	Retrospective analysis. GA: conventional bandage + MLD; GB: alginate bandage + MLD.	CDT: MLD twice/day, 5 days/week (90–120 min). Bandaging (conventional or alginate), education, and exercise included. Evaluations: days 1, 5, 8, 12, 15, 19, 22.	MLD using Vodder technique.	Kuhnke method for arm volume.	Weekday volume decreased (−155.23 mL week 1, −101.02 mL week 2, −61.69 mL week 3). Slight weekend increases. Overall downward trend.
Castro-Sánchez A, Moreno-Lorenzo C, Matarán-Peñarrocha G et al. [35]	*N* = 48 (CG: 24; EG: 24), Mean age: 30–60	RCT. CG: postural measures. EG: elastic containment orthosis + MLD.	Eight-month intervention. MLD 5 days/week followed by containment orthosis. CG received hygiene and postural advice. Evaluated pre- and post-intervention.	MLD using Leduc method.	Measuring tape, EVA, bioimpedance system, Dermatemp infrared thermography, EORTC QLQ-C30.	EG showed significant improvement (*p* < 0.05) in quality of life, extracellular water, function, and mastectomy-side limb volume. CG showed similar gains except in social function.
Listing M, Reibhauer A, Krohn M et al. [4]	*N* = 115 (CG: 57, Mean age: 61.4; EG: 58, Mean age: 57.6)	RCT. CG: usual care. EG: classical massage (back, head, neck).	Private and quiet setting. EG: 30 min massage for 6 weeks. Evaluated pre-treatment, week 5, and 6 weeks post-treatment.	Swedish massage using rose and calendula oils (stroking, kneading, friction, pressure).	SF-8, EORTC QLQ-BR23, GBB, BSF.	Significant reduction in fatigue and physical discomfort (body pain, extremities, breast symptoms) in EG. Reduced apathy and anger, but effects not long-lasting.
Meer T, Noor R, Bashir M et al. [30]	*N* = 36 (GA: 19; GB: 17), Age: 18–60	RCT. GA: MLD. GB: soft tissue mobilization, therapeutic exercise, stretching, strengthening, ROM exercises.	Both groups received 5 sessions/week for 4 weeks. GA: 25 min MLD seated/lying. GB: 20 min limb mobilization. Evaluated pre- and post-intervention.	MLD on shoulder and arm (5–7 movements per section).	EORTC QLQ-C30, QLQ-BR23, DASH, NPRS, PSFS, dynamometer, goniometer.	Both treatments improved QoL, NPRS, MMT, and ROM (*p* > 0.05). MLD more beneficial in DASH and PSFS (*p* < 0.05).
De Baets L, De Groef A, Hagen M et al. [9]	*N* = 48 (CG: 24, Mean age: 52.6; EG: 24, Mean age: 54.8)	Secondary analysis of an RCT. CG: placebo therapy. EG: MFR.	Twelve-week program: weeks 1–8 (2 individual sessions/week), weeks 9–12 (1 session/week). Session: 30 min mobilizations, stretches, massage, exercise. EG received MFR.	MFR techniques on trigger points and fascial adhesions.	Infrared cameras (100 Hz sampling), height index bar.	Significant reduction in scapular protraction (*p* = 0.043) and anterior tilt (*p* = 0.049) in EG. No changes in humerothoracic, trunk, or elbow motion.
De Oliveira M, De Rezende L, Do Amaral M et al. [36]	*N* = 89 (G1: 43, Mean age: 55.6 ± 11.9; G2: 46, Mean age: 56.7 ± 15.1)	Non-randomized controlled clinical trial. G1: MLD. G2: active exercise.	All received postoperative care education; 48 h after surgery, G1 had 40 min MLD sessions and G2 had active group exercise, both 2×/week for 30 days. Evaluated pre-op and 60 days post-op.	MLD with lymph node evacuation and absorption maneuvers.	Flexible measuring tape, goniometer, palpation.	No significant pre–post differences between groups in ROM or circumference.
Freire de Oliveira M, Costa Gurgel M, Pace do Amaral M et al. [26]	*N* = 105 (G1: 52, Mean age: 56.9 ± 11.9; G2: 53, Mean age: 57.3 ± 15.1)	Non-randomized clinical trial. G1: MLD. G2: active exercise.	Postoperative care education; 48 h post-op: G1 received individual MLD, G2 group active exercise, both 40 min, 2×/week, 30 days. Evaluated pre-op and 60 days post-op.	MLD using lymph node evacuation and absorption techniques.	Flexible tape, goniometer, lymphoscintigraphy.	No significant group differences in ROM or circumference; 48% in G1 worsened tracer uptake speed; 13% improved uptake intensity.
Drackley N, Degnim A, Jakub J et al. [37]	*N* = 64, Mean age: 57.6	Pilot study. Manual massage.	Performed in private, relaxing setting with music. Evaluated pre- and post-massage.	Manual massage in area chosen by patient using unscented organic lotion.	VAS, anonymous questionnaire, Likert scale.	Significant post-massage reductions in pain, anxiety, and tension; increased relaxation and general well-being.
Marshall-Mckenna R, Paul L, McFadyen A et al. [31]	*N* = 24 (G1: 14, Mean age: 63.5; G2: 10, Mean age: 51.4)	Pilot study. G1: MFR. G2: usual care.	G1: 4 MFR (1 before radiotherapy, 3 weekly). G2: no physiotherapy. Evaluated at baseline, week 4, and 3 months post-treatment.	MFR techniques.	BMI, digital inclinometer, McGill Pain Questionnaire, DASH, HADS.	G1 showed significant improvement (*p* = 0.001) in all motions. Pain decreased in both groups. DASH improved in G1, no significant difference vs. G2. G2 had higher anxiety and depression.
Mogahed H, Mohamed N, Wahed M [32]	*N* = 40 (CG: 20, Mean age: 43.3 ± 9.28; EG: 20, Mean age: 44.25 ± 9.21)	RCT. CG: traditional shoulder exercises. EG: Cyriax, scapular proprioceptive neuromuscular facilitation + traditional exercises.	Massage for 15 min/session, 3×/week. CG: traditional shoulder exercises 15 min/session, 3×/week (24 sessions total). Evaluated pre- and post-intervention.	Cyriax technique on bicipital groove and serratus anterior.	Goniometer.	EG had significant shoulder ROM improvement and pain reduction (*p* < 0.001) in post-mastectomy adhesive capsulitis compared to CG.
Haghighat S, Lofti-Tokaldany M, Maboudi A et al. [27]	*N* = 137, Mean age: 53.5 ± 10	Descriptive observational clinical trial. CDT.	Daily CDT: 5 days/week (10–15 sessions), including skin care, 45 min light therapy, MLD, exercise, and compression bandaging. Evaluated at first and last session.	MLD using Vodder technique.	Water displacement method, 4-point scale questionnaire.	Significant reductions in edema volume (*p* = 0.03) and lymphedema duration (*p* = 0.002) after phase I CDT.

Note: CG: control group; EG: experimental group; CDT: Complete Decongestive Therapy; MLD: manual lymphatic drainage; AE: active exercise; UC: usual care; MFR: myofascial release; PT: physical therapy; REV: Relative Edema Volume; RVC: Relative Volume Change; TEWL: Transepidermal Water Loss; BCRL: breast cancer-related lymphedema; L-Dex: Lymphedema Index; QOL: Health-Related Quality of Life; SF-8: Quality of Life and Well-Being Survey; VAS: Visual Analog Scale; EORTC QLQ-C30 and EORTC QLQ-BR23: European Organisation for Research and Treatment of Cancer Quality of Life Questionnaires; DASH: Disabilities of the Arm, Shoulder and Hand Scale; NRS: Numerical Rating Scale; PHQ-9: Patient Health Questionnaire; FACT-B+4: Functional Assessment of Cancer Therapy—Breast; VAS: Visual Analogue Scale; PSS-14: Perceived Stress Scale; MMT: Manual Muscle Testing; SPADI: Shoulder Pain and Disability Index; GBB: Giessen Subjective Complaints Inventory; BSF: Berlin Mood Questionnaire; NPRS: Numeric Pain Rating Scale; PSFS: Patient-Specific Functional Scale; ROM: range of motion; BMI: Body Mass Index.

### 3.1. Type of Techniques

Regarding the classification of massage therapy used across the different studies, 14 of the 26 included utilized MLD [11,13,19,20,24,25,26,28,30,33,34,35,36,39], following established methods such as Vodder, Leduc, or Földi techniques, or as part of complex decongestive therapy [12,27,29]. Five studies incorporated MFR [7,8,9,12,31], either as a standalone intervention or combined with stretching and strengthening exercises. Two employed classic manual massage using oils or lotions, applied to various anatomical regions [4,37]. Among less conventional approaches, one study [32] applied the Cyriax technique (deep transverse friction massage) for soft tissue pain relief, while two trials [3,10] focused on foot massage—one utilizing effleurage, petrissage, and superficial friction, and the other implementing reflexology.

In terms of the frequency and duration of the technical sessions, there is heterogeneity in the treatment protocols of the different studies. Most interventions spanned 3–4 weeks, with a five-session-per-week frequency [8,13,15,25,27,30,31,34,39]. Some studies adopted a four-week duration but with fewer total sessions [3,20,24,26,36]. In contrast, Castro-Sánchez et al. [35] conducted an eight-month intervention with daily sessions, while one trial [37] administered only a single treatment session in a controlled environment with relaxing music. The average duration of each session ranged between 15 and 30 min in most studies [3,4,8,9,11,12,13,20,30,31,32]. However, five studies provided sessions exceeding 45 min [25,27,29,34,39], indicating longer treatment durations. In contrast, the study by Arinaga Y. et al. [33] reported sessions lasting only 10 min.

All studies conducted pre- and post-treatment evaluations; however, not all included follow-up assessments to determine whether the intervention’s effects were sustained over time. Seven studies did include follow-up assessments at 1–3 months post-treatment [7,11,13,19,24,31,33]. Two studies [26,36] performed follow-ups 60 days after the intervention, while others [3,4,12,20,25] conducted follow-up measurements several weeks post-treatment. Notably, Baklaci M. et al. [34] conducted weekly assessments, while Cruz-Ramos J. et al. [29] performed daily evaluations.

Table 3 shows the studies classified by type of intervention, and the proportion and frequency of the interventions.

### 3.2. Efficacy of Massage Therapy

Regarding the effects of the massage therapy, seven studies reported significant improvements in the ROM of the affected arm following massage therapy interventions [8,12,20,31,32,35], with three studies documenting sustained long-term improvements [7,11,24]. However, De Oliveira et al. [36] and Freire de Oliveira et al. [26] found no statistically significant differences in ROM compared to groups engaged in active exercise. Similarly, Meer et al. [30] reported no significant differences, though the control group in this study received a multimodal intervention consisting of soft tissue mobilization, therapeutic exercises, stretching, strengthening, and ROM exercises.

Furthermore, de Baets et al. [9] reported a significant reduction in scapular protraction (*p* = 0.043) and anterior tilt (*p* = 0.049) during arm elevation post-intervention using infrared cameras with a 100 Hz sampling rate and a height index elevation bar. Likewise, Rao et al. [8] employed photogrammetry via MB-Ruler version 5.1 software and a digital inclinometer, observing statistically significant improvements in posture (*p* = 0.001) and spinal curvatures.

With respect to lymphatic outcomes, ten of the included studies [12,19,20,25,27,29,33,34,35,39] reported superior reductions in lymphatic volume and arm circumference among participants receiving massage therapy compared to alternative interventions. Comparable results were observed regarding the reduction in arm circumference, with progressive decreases over time in groups receiving any form of massage therapy. Guerero et al. [28] reported significant differences in blood flow velocity when MLD was administered with limb elevation compared to MLD without elevation, though these effects dissipated within 30 min post-intervention.

Quality-of-life measures showed consistent improvement across twelve studies [3,4,7,8,10,19,20,29,31,33,35,37], with particular benefits noted in the domains of depression, anxiety, fatigue, pain perception, and nausea. An exception was reported by one study [25], which found no intergroup differences in DASH scores despite employing an intensive MLD protocol (five weekly sessions over four weeks) supplemented with compression bandaging and health education, contrasting with a control regimen of 12 h daily elastic garment use.

A noteworthy finding emerged from the work of Freire de Oliveira et al. [26], where MLD incorporating nodal evacuation and reabsorption techniques produced dichotomous outcomes: while 48% of participants exhibited reduced radiopharmaceutical uptake velocity, 13% demonstrated enhanced uptake intensity—a result with potential clinical relevance for adjunctive cancer care.

### 3.3. Methodological Quality

The methodological quality assessment results are comprehensively detailed in Table 4. Importantly, negative ratings in this evaluation reflect unreported rather than absent methodological elements, as determined through rigorous article scrutiny.

The methodological quality assessment revealed considerable variability in study rigor across the included investigations. Based on the PEDro scoring criteria, studies were stratified into three quality tiers: ten studies achieved excellent methodological quality (scores 9–10) [3,7,9,10,11,19,25,30,32,35]; eight were classified as good quality (scores 6–8) [4,12,13,20,31,33,36,39]; and seven studies demonstrated fair quality (scores 4–5) [8,24,26,28,29,34,38]. A single outlier study [37] was rated as poor quality (score 0–3), characterized by the absence of a control group, failure to perform intention-to-treat analysis, and a lack of intergroup statistical comparisons. Therefore, a total of 96.15% of the studies showed scores of 5 or more, which implies a methodological quality ranging from fair (appropriate) to excellent, and 69.23% of the articles showed scores between 6 and 10 (good and excellent methodological quality).

Analysis of the study design characteristics revealed that four investigations reported significant between-group differences at baseline [4,8,29,32]. The implementation of blinding procedures was reported in two studies [9,23] and evaluator blinding was reported in seven [7,9,11,19,20,30,31]. PEDro Criterion 8, which assesses follow-up completeness (≥85% of participants included in the final analysis), was met, with the exception of four investigations [4,12,20,24], where dropout rates exceeded this threshold. In addition, three studies omitted intergroup statistical comparisons [13,28,37]. However, all included articles demonstrated satisfactory reporting of eligibility criteria, ensuring transparency in participant selection.

## 4. Discussion

This systematic review aimed to evaluate the efficacy of massage therapy on the symptomatology in women post-mastectomy for breast cancer. While extensive literature exists on breast cancer management, research specifically examining massage therapy interventions remains comparatively limited. This paucity contrasts sharply with the substantial body of evidence supporting therapeutic exercise—a discrepancy that may reflect shifting clinical trends favoring exercise-based interventions over manual therapies. Such preference may stem from an increasing awareness of work-related musculoskeletal injuries, particularly hand pathologies, among physiotherapists performing manual techniques [40].

Current evidence demonstrates that both aerobic and resistance-based exercise protocols effectively manage breast cancer-related lymphedema without adverse effects. When integrated with MLD, these interventions demonstrate synergistic benefits, including enhanced muscular strength and endurance, reduced pain and limb volume, and improved upper extremity functionality [40].

The selection criteria for this review (ages 45–64 years) were informed by epidemiological data [1,2,41,42] showing that (1) breast cancer incidence rises sharply with age and hormonal change during menopause (ref. [2,41,42]); cases before age 25 are exceptionally rare; and (3) peak incidence occurs in later adulthood. This age range consequently represents a critical risk period, reflected in international screening guidelines that typically initiate routine mammography at age 50 [2,41].

A total of 17 of the 26 included studies [11,12,13,19,20,24,25,26,28,29,30,33,34,35,36,38,39] utilized MLD as the primary intervention, predominantly administered by professionals certified in established methods (Vodder, Leduc, or Földi techniques) [25,28,29,35,38,39]. This clinical preference is supported by robust physiological evidence indicating that MLD enhances lymphatic vessel contractility, promotes protein reabsorption, and alleviates microlymphatic hypertension [43]. Importantly, current oncological research confirms that metastasis represents a complex biological process unaffected by mechanical manipulation inherent to MLD techniques [44].

Notably, Baklaci et al. [34] implemented a patient-administered MLD protocol, with participants receiving professional training to ensure proper self-application throughout the study duration. Alternative approaches incorporating MFR [7,8,12,31] consistently demonstrated multidimensional quality of life improvements, particularly in pain perception, fatigue levels, and depression and anxiety measures. The comparative study by Serra-Añó et al. [7] revealed differential outcomes between MLD and MFR: while both interventions showed comparable efficacy, MLD produced superior emotional well-being outcomes, whereas MFR exhibited more sustained effects on pain reduction and functional improvement. These findings underscore that clinical effectiveness depends on proper technique execution by skilled practitioners.

Mogahed et al. [32] demonstrated that brief 15 min interventions using the Cyriax technique produced significant improvements in both pain scores and range of motion parameters. This suggests that abbreviated treatment protocols may improve patient compliance, particularly for working women facing time constraints.

The reviewed studies demonstrated variability in treatment settings, with only three investigations [4,10,37] explicitly reporting the use of private clinical or hospital rooms for therapy administration. One notable exception [33] employed a self-administered MLD protocol, suggesting home-based treatment implementation. While patient-delivered interventions produced less robust outcomes than professional applications, they nevertheless resulted in measurable improvements across several parameters, including quality of life metrics, transdermal water loss, and lymphedema severity. These findings suggest that properly instructed self-care regimens may represent a potentially valuable adjunct to conventional treatment, meriting further research to evaluate their role in optimizing long-term outcomes and preventing symptom recurrence.

The impact of massage therapy on quality of life emerged as a consistent finding across multiple studies. Dllaveri et al. [45] reported significant stress reduction following traditional massage interventions in breast cancer patients, while Krohn et al. [46] observed differential effects with notable improvements in depression and anxiety but non-significant changes in stress or mood outcomes. Comparative analysis revealed disease-specific response patterns, with one study [47] documenting that anxiety decreased more in women with breast cancer than in those with other types of cancer, while depression, fatigue, sleep disorders, and emotional well-being improved to a lesser extent in the breast cancer group.

Regarding the results related to methodological quality, we can highlight that even though negative ratings on the PEDro Scale reflected unreported rather than absent methodological elements, as determined through rigorous article scrutiny, a total of 96.15% of the studies showed scores of 5 or more, which implies a methodological quality ranging from fair (appropriate) to excellent, and 69.23% of the articles showed scores between 6 and 10 (good and excellent). These results support the interpretation of the improvements achieved by the interventions. However, we consider that we need to be cautious due to the existence of non-reported data for some of the scale’s items, such as blinding, sample size, or intention-to-treat analysis.

### Limitations

Several important limitations should be considered when interpreting these findings. First, the inclusion of multiple studies lacking control groups [8,29,34,37,38] precludes robust comparisons between massage therapy and either alternative interventions or placebo conditions. This methodological constraint substantially limits the generalizability of results, as the majority of included studies employed comparative treatment designs rather than controlled experimental protocols.

Second, the current evidence base suffers from significant heterogeneity in treatment parameters, with only one study [7] providing direct comparisons between different massage techniques. The substantial variability in treatment frequency and session duration across studies further complicates efforts to identify optimal therapeutic protocols or establish dose–response relationships. On the other hand, although most of the research is indexed mainly in English-language databases, it would be interesting to analyze publications in other languages, as well as whether there is research with a smaller sample than that included in this study that provides relevant data on the topic under study.

Finally, while this review focused specifically on post-mastectomy patients, the aggregation of data across varying tumor stages and lymphedema phases may obscure important clinical distinctions. Future research should employ stratified designs to examine whether treatment efficacy varies according to these clinical factors. Additionally, further investigation is warranted into the potential adverse effects of massage techniques in women who have undergone mastectomy, with or without prostheses, as well as the emotional benefits these interventions may provide to patients.

## 5. Conclusions

The current evidence synthesis suggests that various massage therapy modalities—including MLD, MFR, Cyriax technique, foot massage, and classical massage—demonstrate therapeutic efficacy comparable to conventional active exercise and standard treatments in post-mastectomy care with fair to excellent methodological quality. These interventions have shown consistent benefits across multiple domains, particularly in enhancing upper limb mobility through improved range of motion (in flexion, extension, abduction, and adduction) and reducing both lymphedema volume and overall arm circumference. Importantly, massage therapies appear to offer protective effects against post-mastectomy lymphedema development.

Beyond physical outcomes, massage interventions yield significant psychosocial benefits, effectively alleviating common comorbidities such as anxiety and depression while mitigating cancer-related symptoms, including fatigue, pain sensitivity, nausea, and muscular tension. The passive nature of massage therapy provides additional value by promoting psychological relaxation and stress reduction, aspects particularly valuable during the demanding recovery process.

Therefore, based on the results of the studies analyzed, massage therapy interventions may represent a suitable complementary approach to post-mastectomy breast cancer treatment. However, despite the existing body of evidence, further high-quality studies with long-term follow up and homogeneous research, as well as studies with a focus on identifying optimal massage techniques and treatment duration, are needed to strengthen and consolidate these findings.

## Figures and Tables

**Table 1:** Syntax of combined descriptors in the scientific database search.

Database	Syntax
PubMed	‘Mastectomy AND breast neoplasms AND massage’; ‘Mastectomy AND breast neoplasms AND manual lymphatic drainage’; ‘myofascial release therapy AND breast neoplasms’
Cochrane	‘Mastectomy AND breast neoplasms AND massage’; ‘Mastectomy AND breast neoplasms AND manual lymphatic drainage’; ‘mastectomy AND myofascial release’
PEDRO	‘Mastectomy AND massage’; ‘Mastectomy AND manual lymphatic drainage’; ‘mastectomy AND myofascial release’
Dialnet	‘Mastectomy AND breast neoplasms AND massage’; ‘Mastectomy AND manual lymphatic drainage’; ‘mastectomy AND myofascial release’
ScienceDirect	‘Mastectomy AND massage’; ‘Mastectomy AND manual lymphatic drainage’; ‘mastectomy AND myofascial release’
Scopus	‘Mastectomy AND breast neoplasms AND massage’; ‘Mastectomy AND breast neoplasms AND manual lymphatic drainage’; ‘mastectomy AND myofascial release’

**Table 3:** Intervention types, session frequency, and duration.

Authors	Intervention Types	Session Frequency	Duration
Arinaga Y, Piller N, Sato F et al. [33]	MLD	Daily	6 weeks
Baklaci M, Eyigör S, Tanlgör G et al. [34]	MLD	5 days/week	Not specified
Xiong Q, Luo F, Zhan J et al. [11]	MLD	Daily	3 months
Uzkeser H, Karatay S, Erdemce B et al. [19]	MLD	5 days/week	3 weeks
Guerrero R, das Neves L, Guirro R et al. [28]	MLD	Not specified	Not specified
Oliveira M, Gurgel M, Amorim B et al. [24]	MLD	2 days/week	30 days
Martín M, Hernández M, Avendaño C et al. [13]	MLD	Not specified	4 weeks
Dayes I, Whelan T, Julian J et al. [25]	MLD	5 days/week	4 weeks
Cho Y, Do J, Jung S et al. [20]	MLD	3 days/week	4 weeks
Cruz-Ramos J, Cedeño-Meza A, Bernal-Gallardo J et al. [29]	MLD	Daily	5 days
Kasseroller R, Brenner E [39]	MLD	2 days/week	5 days
Castro-Sánchez A, Moreno-Lorenzo C, Matarán-Peñarrocha G et al. [35]	MLD	5 days/week	8 months
Meer T, Noor R, Bashir M et al. [30]	MLD	5 days/week.	4 weeks
De Oliveira M, De Rezende L, Do Amaral M et al. [36]	MLD	2 days/week	Not specified
Freire de Oliveira M, Costa Gurgel M, Pace do Amaral M et al. [26]	MLD	2 days/week	30 days
Haghighat S, Lofti-Tokaldany M, Maboudi A et al. [27]	MLD	5 days/week	10–15 sessions
Serra-Añó, Inglés M, Bou-Catalá et al. [7]	MFR	Not specified	Not specified
Kim Y, Park E, Lee H [12]	MFR	Not specified	Not specified
Rao M, Pattanshetty R [8]	MFR	4 sessions/week	3 weeks
De Baets L, De Groef A, Hagen M et al. [9]	MFR	1 or 2 sessions/week	12 weeks
Marshall-Mckenna R, Paul L, McFadyen A et al. [31]	MFR	3 sessions/week	4 weeks
Dion L, Engen D, Lemaine V et al. [10]	Swedish massage	3 days	Not specified
Listing M, Reibhauer A, Krohn M et al. [4]	Swedish massage	Not specified	6 weeks
Demirci P, Tasci S, Öztunç G [3]	Other massage techniques	2 sessions/week	3 weeks
Drackley N, Degnim A, Jakub J et al. [37]	Other massage techniques	3 sessions/week	Not specified
Mogahed H, Mohamed N, Wahed M [32]	Other massage techniques	3 sessions/week.	4 weeks

MLD: manual lymphatic drainage; MFR: myofascial release.

**Table 4:** Evaluation of the methodological quality of the studies (PEDro).

Study	Criteria												
	1	2	3	4	5	6	7	8	9	10	11	Score	Results
Arinaga Y et al. [33]	Y	Y	N	Y	N	N	N	Y	Y	Y	Y	7	Good
Baklaci M et al. [34]	Y	N	N	Y	N	N	N	Y	Y	Y	N	5	Fair
Xiong Q et al. [11]	Y	Y	N	Y	N	N	Y	Y	Y	Y	Y	9	Excellent
Uzkeser H et al. [19]	Y	Y	Y	Y	Y	N	Y	Y	Y	Y	Y	10	Excellent
Guerero R et al. [28]	Y	Y	N	Y	N	N	N	Y	Y	N	N	5	Fair
De Oliveira M et al. [24]	Y	N	N	Y	N	N	N	N	Y	Y	Y	5	Fair
Martín M et al. [13]	Y	Y	N	Y	Y	N	N	Y	Y	N	N	6	Good
Dayes I et al. [25]	Y	Y	N	Y	Y	Y	N	Y	Y	Y	Y	9	Excellent
Cho Y et al. [20]	Y	Y	N	Y	Y	N	Y	N	Y	Y	Y	8	Good
Serra-Añó et al. [7]	Y	Y	Y	Y	Y	N	Y	Y	Y	Y	Y	10	Excellent
Dion L et al. [10]	Y	Y	Y	Y	Y	N	N	Y	Y	Y	Y	9	Excellent
Kim Y, Park E, Lee H [12]	Y	Y	Y	Y	Y	N	N	N	Y	Y	Y	8	Good
Cruz-Ramos J et al. [29]	Y	N	N	N	N	N	N	Y	Y	Y	Y	5	Fair
Demirci P, Tasci S, Öztunç G [3]	Y	Y	Y	Y	Y	N	N	Y	Y	Y	Y	9	Excellent
Rao M, Pattanshetty R [8]	Y	N	N	N	N	N	N	Y	Y	Y	Y	5	Fair
Kasseroller R, Brenner E [39]	Y	Y	Y	Y	N	N	N	Y	Y	Y	N	7	Good
Castro-Sánchez A et al. [35]	Y	Y	Y	Y	Y	N	N	Y	Y	Y	Y	9	Excellent
Listing M et al. [4]	Y	Y	Y	N	Y	N	N	N	Y	Y	Y	7	Good
Meer T et al. [30]	Y	Y	Y	Y	Y	N	Y	Y	Y	Y	Y	10	Excellent
De Baets L et al. [9]	Y	Y	Y	Y	Y	Y	Y	Y	Y	Y	N	10	Excellent
De Oliveira M et al. [36]	Y	N	N	Y	N	N	N	Y	Y	Y	Y	6	Good
Freire de Oliveira M et al. [26]	Y	N	N	Y	N	N	N	Y	Y	Y	N	5	Fair
Drackley N et al. [37]	Y	N	N	Y	N	N	N	Y	N	N	N	3	Poor
Marshall-Mckenna R et al. [31]	Y	Y	N	N	Y	N	Y	Y	Y	Y	Y	8	Good
Mogahed H, Mohamed N, Wahed M [32]	Y	Y	Y	Y	Y	N	N	Y	Y	Y	Y	9	Excellent
Haghighat S et al. [38]	Y	N	N	Y	N	N	N	Y	Y	Y	N	5	Fair

Y: met criteria; N: did not meet criteria.

## Data Availability

Data is available upon reasonable request to the authors.
